# The Pathology and Treatment of Ulcers of the Lower Extremity

**Published:** 1882-01

**Authors:** George H. Rohe

**Affiliations:** Baltimore, Md., Clinical Professor of Dermatology, College of Physicians and Surgeons, Baltimore


### THE PATHOLOGY AND TREATMENT OF ULCERS OF THE LOWER EXTREMITIES.

By GEORGE H. ROHE, M.D., Baltimore, Md.,
Clinical Professor of Dermatology, College of Physicians and Surgeons, Baltimore.

In accordance with the present doctrines of pathology, an ulcer may be defined as " a loss of tissue extending into and through the tissue of the corium, accompanied by a secretion of pus—as a rule, not of the sort termed laudable." In this process the tissue-waste or necrosis keeps pace or exceeds the biosis or formative action, and thus prevents or delays healing. When healing does take place it is by granulation and cicatrization, leaving a scar to mark the site of the ulceration.

Normal tissue is never the seat of ulceration; there must necessarily have gone before an inflammatory infiltration or other new formation. The general causes of ulceration are traumatism, whether caused by contusion, laceration, scratching or chemical irritants—or abnormal conditions having their seat in the tissues themselves; varicosity of the veins, atheromatous degeneration of the arteries, and chronic inflammation.

Certain constitutional conditions and new formations are accompanied by ulcerations, such as syphilis and scrofula, carcinoma and lupus.

The clinical character of an ulcer finds expression in its base, its borders, its secretion and its course.

The base of an ulcer consists of a somewhat uneven surface, at a greater or less depth below the surface of the skin, covered with pus and necrosed tissue, and studded here and there with granulations. The edge or border is more or less distinctly defined against the base, and may be raised above the surface of the surrounding integument. It may slope gradually from the base or rise abruptly It may be undermined, overhanging the circumference of the base, or present an elevated ridge running around the excavation It may finally be smooth or ragged. In very chronic ulcers the border is sometimes thickly infiltrated and hard—the so called " callous " ulcer.

The pus secreted on the surface of the ulcer generally differs more or less from that termed "laudable" by surgeons. It has received various names, such as sanious, ichorous, serous, flaky, strumous, etc. It may have no perceptible odor, but is usually offensive. If left to desiccate upon the surface of the ulcer it forms, according to its consistence, either thick yellowish or brownish scabs, or thin, shiny crusts.

In the beginning an ulcer is generally circular. As it progresses it may assume various shapes; thus it may become oval, kidney-shaped, sinuous, or of an irregular figure.

Ulcers are generally more or less painful, at times, however, they appear to be almost entirely devoid of sensation.

The course of an ulcer may be divided into a stage of progression and of retrogression, or in other words, a stage of destruction and a stage of construction or repair. During the stage of destruction the necrosis of tissue is in excess of the formative action, and it is only when the latter gains the upper hand that the process of healing begins. The normal progress of the stage of repair is as follows: The inflammation and swelling of the tissues, the pain and secretion of pus decrease, bright red, fleshy points (granulations) spring up upon the base and around the border of the ulcer. The ulcer becomes more shallow, and the border more continuous between the base and the surrounding skin. The formation of the new epidermis is indicated by the advance of a bluish-white or white pellicle from the edge of the ulcer toward the centre. When the centre is reached cicatrization is completed and the ulcer healed.

Deviations from this typical behaviour may occur, and we then have—in the destructive stage—phagedena, gangrene and diphtheria occurring; while the repair may be delayed by the formation of defective granulations, as happens in the so-called atonic ulcers, or the granulations may become too luxuriant and hydropic, and we then have the so-called "proud flesh." Taking as the type of an ulcer, that which occurs upon the leg, and which is generally termed "the varicose ulcer," let us study its evolution and progress.

Persons who are, by the necessities of their occupation, compelled to stand a great deal, frequently have an enlargement or dilatation of the veins of the lower extremities accompanied by more or less itching. This itching is the result of frequently repeated gentle irritation of the peripheral terminations of the nerves, by the stagnated blood in the dilated veins and capillaries. The itching gives rise to scratching, and, perhaps, when our attention is first called to the part we find a moderate degree of eczematous inflammation, the result of the scratching.

Small hemorrhagic spots may also be noticed here and there in the skin of the affected limb, indicating that rupture of the capillaries has already taken place.

With the exception of these minute petechiæ, and the papular and pustular expressions of the eczema, the skin appears normal. If such persons are able to take care of themselves: that is, quit their regular occupation or assume the recumbent posture, these effects soon disappear; even if small superficial losses of substance—ulcerations—have already taken place. If, however, on the contrary, the causes mentioned continue or are intensified, the disturbances of nutrition keep pace with them, and in the course of months or years—the losses of substance in the meanwhile becoming larger and more permanent—the leg presents the condition spoken of as varicose ulceration.

"Varicose ulcer," it will be seen, is not a very accurate designation, because the ulcer itself is not varicose at all. It is the result of a varicose or dilated condition of the veins of the part.

Various other disturbances occur at times while those just described have been going on, and we may have lymphangitis, erysipelas and abscesses forming, which still further intensify the ulcerating processes. Finally, these multiform perversions of nutrition are followed by permanent dilatation of veins and capillaries, œdema of the tissues, etc., which, in the course of time, have as a consequence, hyperplasia and degeneration of the connective tissue, and even of the muscles and bones. In short we have developed a real elephantiasis of the leg.

Beginning thus, with trifling symptoms, the morbid action continuing throughout a number of months or years produces a singularly complex series of symptoms, the most prominent of which is the fully developed ulcer. The appearances are then somewhat as follows: The integument of the leg, beginning at the tibial spine, presents, at intervals, portions which are blueish or brownish red, smooth and shiny; other portions are covered with small whitish scales, or thickened patches of fissure epidermis. Here and there are thin, whitish scars, the remains of previous ulcers. Otherwise the skin is thin, and tightly stretched over the border of the tibia. At times, however, the portions immediately above the ankle are much thickened and covered with warty excrescences. Within this territory exist one or more, isolated or confluent, irregularly deep ulcers, with callous (gristly) borders, which secrete a thin and viscid matter, generally of offensive odor. The granulations are usually few in number and bleed easily when touched. These ulcers are often of considerable extent. They sometimes form an irregular band extending two-thirds the circumference of the limb. They nearly always occupy the lower third and anterior aspect of the leg. The explanation of this situation of predilection has been given by Hilton in his lectures on "Rest and Pain." It will be remembered that there are two systems of veins in the leg and foot—the superficial and deep. The superficial veins, being the two *saphena*—external and internal—arise from the dorsal venous arch, and ascend the leg and thigh, the internal saphena, passing through the *fascia lata* near the pubic insertion of Poupart's ligament, unites with the deep femoral vein. The external saphena passing behind the external malleolus and over the gastrocnemius muscle empties into the popliteal vein in the popliteal space. The deep veins lie between the muscles accompanying the arteries. Now, at the ankle-joint there is a free anastomosis between the superficial and deep veins, enabling the latter to relieve any extra pressure of blood in the external veins at that part, while two inches above the joint the communication is less free, and when any extraordinary pressure occurs here the veins, not being able to discharge their contents rapidly enough, dilate and give way. Another cause is the small quantity of subcutaneous connective tissue at this point, and the tense condition of the skin passing across the edge of the tibia.

All ulcers occurring upon the lower extremities are not due to varicosities of the veins. Some are due to chronic eczema; others to traumatism; and still others to the ulcerative breaking down of new formations — lupus, cancer and syphilis. The diagnosis is in most cases easy, however. Lupus always begins early in life and progresses slowly. In cancer, the peculiar hard infiltration beyond the edges is characteristic; while in syphilis, the history of the constitutional disease can generally be made out. An important diagnostic mark between syphilitic and varicose ulcers has been pointed out by Mr. Maunder, an English surgeon. It is this: Syphilitic ulcers of the leg usually are situated on the upper half of the limb, and are multiple; varicose ulcers, on the other hand, are on the lower half, and preferably just above the malleoli, and are most often solitary.

The treatment of ulcers requires, besides an accurate knowledge of their pathology and the remedies to be used, especially the quality of patience. The cure of a chronic ulcer of the leg is not a question of days, but of weeks; sometimes even of months. Careful and patient attention are therefore necessary, and unless the practitioner is willing to give these in any case about which he may be consulted, he had better not meddle with it. The objects of our remedial measures are three:

1. To diminish or remove inflammation and its consequences.

2. To promote the casting off of the necrosed tissue; and,

3. To promote the cicatrization and remove everything calculated to hinder this result.

These objects are attained in various ways. If the patient can assume the recumbent position, almost any method of treatment will suffice to cure the ulcer. But very few patients of the class affected with these ulcers can afford to do this. Some method of treatment must be adopted in their case that will permit them to pursue their customary occupations. In bandaging or strapping we possess such a method, and when properly applied the majority of cases will get well under its influence.

The method of strapping ulcers with adhesive plaster was introduced by Thomas Baynton, an English surgeon, about the beginning of the present century. It consists in the application of strips of adhesive plaster, ½ to 2 inches wide, and long enough to go completely around the limb and cross for some distance over the ulcer. It is better to make the method antiseptic, which can be done by saturating a piece of lint in carbolic or boracic lotion, and covering the ulcer with it, applying the strips over this. A muslin or flannel roller should then be evenly and firmly applied from the toes to the knee. This dressing affords admirable support to the sides of the ulcer and to the enlarged veins, and under its use, many, indeed most ulcers will rapidly get well. If applied with antiseptic precautions this strapping may be left on four or five days; otherwise it will have to be removed every other day at the farthest.

The most convenient, and at the same time, most comfortable for the patient, is bandaging the limb with a roller bandage made of "domette" flannel. A bandage of this kind should be from 2–2½ inches wide, and six to eight yards long. Having dressed the ulcer with simple cerate, oxide of zinc ointment, carbolic ointment, oil or lotion, or what is perhaps better, iodoform ointment (ʒi—℥i), the roller is applied in even turns from the toes to the knee, exercising firm but equable pressure from first to last. The dressing should be changed every second or third day. In cases of old indolent ulcers, the application of balsam of Peru or copaiba will have a very happy effect. Where the base of the ulcer is unhealthy, or the granulations exuberant, an application of silver nitrate, either in pencil or saturated solution, will produce a satisfactory alteration in the character of the process.

Sometimes the border of the ulcer presents a hard, almost cartilaginous feel. In these cases, the remedy is to take a scalpel and make a number of incisions into the thickened edge in a radiating manner, or the incisions may encircle the ulcer as if to enucleate it. These radiating incisions are also useful when the border is inflamed, as the discharge of blood relieves the engorgement. If the ulcerated surface is too large to heal by cicatrization, the operation of skin-grafting may be resorted to. This is troublesome and very rarely successful.

